# Copper toxicity on *Eisenia fetida* in a vineyard soil: a combined study with standard tests, genotoxicity assessment and gut metagenomic analysis

**DOI:** 10.1007/s11356-024-31946-6

**Published:** 2024-01-19

**Authors:** Enrica Marini, Arianna De Bernardi, Francesca Tagliabue, Cristiano Casucci, Luca Tiano, Fabio Marcheggiani, Filippo Vaccari, Eren Taskin, Edoardo Puglisi, Gianluca Brunetti, Costantino Vischetti

**Affiliations:** 1 Department of Agricultural, Food and Environmental Sciences, Polytechnic University of Marche, Via Brecce Bianche, 60131 Ancona, Italy; 2 Department of Life and Environmental Sciences, Polytechnic University of Marche, Via Brecce Bianche, 60131 Ancona, Italy; 3 Department for Sustainable Food Process, Faculty of Agriculture, Food and Environmental Sciences, Università Cattolica del Sacro Cuore, Via Emilia Parmense 84, 29122 Piacenza, Italy; 4 Future Industries Institute, University of South Australia, Mawson Lakes Boulevard, Mawson Lakes, South Australia SA5095 Australia

**Keywords:** Ecotoxicology, Earthworms, Coelomocytes, Single cell gel electrophoresis, Comet assay, Next-generation sequencing, Copper, Vineyard soil

## Abstract

Copper (Cu) toxicity is a pressing concern for several soils, especially in organic viticulture. The objective of this work was to assess Cu toxicity on the non-target organism *Eisenia fetida*, employing both traditional and novel tools for early identification of Cu-induced damages. In addition to traditional tests like avoidance and reproductive toxicity experiments, other tests such as the single cell gel electrophoresis (SCGE) and gut microbiome analysis were evaluated to identify early and more sensitive pollution biomarkers. Four sub-lethal Cu concentrations were studied, and the results showed strong dose-dependent responses by the earthworm avoidance test and the exceeding of habitat threshold limit at the higher Cu doses. An inverse proportionality was observed between reproductive output and soil Cu concentration. Bioaccumulation was not detected in earthworms; soil concentrations of potentially bioavailable Cu were not affected by *E. fetida* presence or by time. On the contrary, the SCGE test revealed dose-dependent genotoxicity for the ‘tail length’ parameter already at the second day of Cu exposition. Gut microbiome analysis a modulation of microbial composition, with the most aboundant families being *Pectobateriaceae*, *Comamonadaceae* and *Microscillaceae*. *Bacillaceae* increased over time and showed adaptability to copper up to 165 mg/kg, while at the highest dose even the sensitive *Acetobacteriaceae* family was affected. The research provided new insights into the ecotoxicity of Cu sub-lethal doses highlighting both alterations at earthworms’ cellular level and changes in their gut microbiota.

**Supplementary Information:**

The online version contains supplementary material available at 10.1007/s11356-024-31946-6.

## Introduction

Viticulture is a crucial agricultural sector in the Mediterranean region, with Italy, France and Spain together accounting for around 60% of the World wine production (Hall and Mitchell 2000). In vineyards, the use of copper (Cu) compounds against fungal diseases has a history dating back more than a hundred years (Merry et al. 1983). These long-term applications resulted in several cases of Cu accumulation in soils, which may also have adverse effects on non-target species (Helling et al. 2000; Maboeta et al. 2002; Van Zwieten et al. 2004). The replacement of copper-based pesticides is still under discussion at the European Union level (European Commission 2018), and most of the studies are dated back to more than 20 years ago, when the annual maximum permissible dose of 4 kg/ha was not yet in force for organic viticulture (EFSA et al. 2018). Novel experiments on the impacts of copper in organic viticulture, especially on soil organisms, are thus highly recommended (Karimi et al. 2021).

Although Cu is an essential micronutrient for plants, it is often toxic to other soil organisms (Tarradellas et al. 1996) and can induce drastic changes in the microbial communities in soils as well as in the intestines of soil organisms (Šrut et al. 2019). Copper-based fungicides can indeed negatively affects earthworms and microbial communities, with the consequential impairment of important soil functions (FAO and ITPS 2017; Merrington et al. 2002).

Earthworms are key members of the biota of soils, where they carry out invaluable activities for soil health and fertility (Edwards and Bohlen 1996). These annelids interact with the bacterial communities and can also modify the speciation and availability of metals through their digging activity and by ingesting soil particles (Sizmur and Hodson 2009). Earthworms are considered good indicators of the occurrence and toxicity of both inorganic and organic pollutants (Jager et al. 2003; Vijver et al. 2003); for this reason, *Eisenia fetida* (*E. fetida*) is employed in standard ecotoxicity tests (ISO-17512–1, 2008; OECD 2016, 1984). Nevertheless, there are still some uncertainties regarding the effects that Cu excess can have on earthworms’ reproduction (fertility parameters) and development (biomass growth) and their relation with other endpoints such as oxidative damages (Clasen et al. 2021).

The relevance of the investigation on the earthworms’ gut microbiome to understand the impacts of environmental pollutants, such as metals and pesticides, on the soils’ biological quality has been the subject of a number of recent works (Jin et al. 2017; Šrut et al. 2019; Vischetti et al. 2020; Yausheva et al. 2016). Pollutants can indeed affect the earthworms’ gut microbiota, leading to a disequilibrium in their immune system, constituted by immune cells called coelomocytes (Jin et al. 2017; Swart et al. 2020a). Furthermore, since some pollutants can induce genetic alterations due to stress that can be currently measurable in non-target organisms even at sub-lethal level, we are proposing that innovative tools such as the genotoxicity analysis with single cell gel electrophoresis assay (comet assay) can be employed to evaluate induced stresses in ecotoxicological studies (Li et al. 2009; Lourenço et al. 2011; Mincarelli et al. 2016; Tice et al. 2000).

In the present work, a vineyard soil with four sub-lethal copper concentrations was evaluated, apart from the standard earthworms’ avoidance and reproduction tests, with new toxicity tests to evaluate both changes in gut microbial community and *E. fetida* DNA damages. Cu accumulation was also monitored in both soils and earthworms in order to correlate toxicological dynamics with the occurring environmental conditions. The main goal of the work was to provide early and more sensitive risk assessment tools to implement the understanding of Cu impacts on organic vineyard soil ecosystems.

## Materials and methods

### Earthworms

*E. fetida* earthworms were reared in controlled laboratory conditions (at 20 ± 1 °C in the dark) using commercial potting soil as substrate and organic oats and vegetables as food. Ten adults, with a well-developed clitellum and wet body weights of at least 300 mg, were used as each replicate in all the experiments; their acclimatisation was performed respectively in the same soil used in each of the experiments (Li et al. 2018; Zhang et al. 2019; Zou et al. 2018). Specifically, the untreated vineyard soil was used for the acclimatisation of all earthworms except for those destined for artificial soil, which were instead acclimated in the standard OECD soil.

### Soils

The soil used in all the experiments was collected from the top soil (0–20 cm depth) of an Italian vineyard managed with organic agricultural practices; after collection it was air-dried, homogenised and sieved at 2 mm. The untreated vineyard soil was a silty clay loam soil (19.6% sand, 48.7% silt and 31.7% clay content), characterised by a slightly alkaline pH (pH_(H2O)_ = 7.79), and an organic matter content of 1.4%. The total copper concentration measured in this soil was 55 ± 1.66 mg/kg, and it was coded as FC (field concentration). To test other copper concentrations in a geometric series, the FC starting soil was further spiked in the lab to reach Cu copper concentrations of 110 ± 1.30, 165 ± 1.95 and 220 ± 1.28 mg/kg, respectively labelled as 110, 165 and 220.

Apart from the untreated soil (FC), these three Cu concentrations were selected taking into account both the 200 mg/kg threshold concentration set for Cu contamination of Italian agricultural soils (Annex 2, Decree No. 46; Italian Official Gazette 2019), and the 155 mg/kg threshold value indicated for Cu-sulphate LC_50_ (*E. fetida* 50% lethal concentration), as reported in the Pesticides Properties DataBase (PPDB). These potential sub-lethal Cu concentrations were obtained by spiking the untreated soil (FC) by mixing with fresh solutions of a commercial Cu sulphate-based fungicide (Siaram 20 WG) into the soil, as recommended in the OECD guideline 222 (OECD 2016).

In addition to the unspiked soil, a negative control was prepared using an artificial soil (ART) according to the standard OECD guideline 207 (OECD 1984). Total Cu concentration in the artificial soil was 7 ± 1.07 mg/kg.

In all experiments, the soils were kept at a moisture content of 27% (corresponding to 42.8 and 43.3 of the water holding capacity, respectively, for ART and FC soil) with deionized water was reached and maintained by weighing the containers periodically. The quantity of water used in the spiking procedure was counted in the contribution necessary to reach this humidity rate.

### Avoidance test

A “dual control” test was initially performed using the untreated soil FC or the artificial soil (ART) in both sides of the test chamber to check that earthworms do not tend to prefer one of the two sections when they are filled with the same substrate (Hund-Rinke and Wiechering 2001; Yeardley et al. 1996). The avoidance test was then conducted in five replicates per each treatment (110, 165 and 220) with the two-chamber design, as described by ISO 17512–1 (ISO, 2008) to find whether the earthworm *E. fetida* avoids contaminated soils (García-Santos and Keller-Forrer 2011; Jordaan et al. 2012; Martínez Morcillo et al. 2013). In each replicate, one-half of the box was filled with 250 g of dry weight of the copper-contaminated substrates of 110, 165 or 220 mg/kg, and the other half was filled with the same quantity of the top-soil sampled from vineyard used as a control (FC); ten earthworms were placed in the middle of the box. After 2 days, the earthworms in both chambers were counted. The results of the avoidance test are expressed as the net response (*NR*) according to ISO-17512–1 (ISO, 2008):

$$NR(\%)=\left\lfloor\left(C-T\right)\div N\right\rfloor\times100$$

where *C* and *T* are the number of worms in the FC soil and the contaminated soil, respectively; *N* is the total number of worms in each box.

Although avoidance is a sensitive and easy to understand method, alone it cannot effectively describe the ecotoxicological effects of a xenobiotic (Pelosi et al. 2014); for this reason, two further tests were conducted.

### Reproductive test

Copper impacts on earthworms’ reproductive output, i.e. a number of cocoons and juveniles in each replicate, and other sub-lethal endpoints, such as behaviour alterations and weekly body changes, were assessed through a reproductive toxicity test following the OECD guideline 222 (OECD 2016).

All treatments (FC, 110, 165, 220 and ART) were tested in three replicates, each replicate consisting of 500 g of dry soil inoculated individually with ten mature earthworms and kept for 56 days in a controlled environment with constant temperature and soil water content (20 ± 1 °C; 27% w/w). The weights of all adult earthworms in each replicate were recorded weekly, including any unusual behaviour and morphology anomalies. After 28 days, only adults were removed from the containers, while soils containing juveniles and cocoons were incubated for further 4 weeks. On the 56th day, a number of juveniles and the cocoons in each replicate were recorded by hand-sorting. The adults’ ponderal growth rate (*GR*), expressed as percentage over 4 weeks, was calculated as follows:

$$GR \left(\%\right)=[\left({W}_{t }-{W}_{0}\right)\div {W}_{0}]\times 100$$

where *W*_0_ was the initial average weight of earthworms, and *W*_t_ was the average weight of earthworms on day 28. A positive rate means growth stimulation, while a negative rate indicates growth inhibition (Xie et al. 2013).

### Genotoxicity and gut microbiome test

Another similar experiment was set up to gain more detailed information on toxicity; in particular, this trial aimed to evaluate the potential Cu bioaccumulation in earthworms, to investigate the genotoxicity, i.e. the potential damages on the earthworms’ DNA using the SCGE approach, and to estimate possible alterations of the earthworms’ gut microbiome.

Similar to the reproductive test, triplicates of every treatment (FC, 110, 165, 220 and ART) consisting of 500 g of dry soil per replicate were incubated for 4 weeks with 10 earthworms in the same homeostatic conditions mentioned before (20 ± 1 °C and 27% water content). In this case, a further test without adding earthworms was also carried out in parallel to evaluate any potential differences in Cu soil concentration due to the *E. fetida* bioaccumulation. Samplings for bioaccumulation study were performed both at the beginning and at the end of the test, respectively after 2 and 28 days of incubation. At each sampling time and for every treatment, three soil samples, one for each replicate pot, were analysed to assess Cu concentrations. Contextually, three earthworms, one for each replicate pot, were sacrificed to measure the bioaccumulation factor.

Total Cu concentration in soils was determined by the acid digestion method described by Kasassi et al. (2008) with some modifications: 0.5 g of dry soil was predigested overnight with 2 mL of H_2_O_2_ (30% v/v) and then acid digested for 15 h with 7 mL of concentrated HNO_3_ (65% v/v) in a hot (> 85 °C) floating water bath. The potentially bioavailable Cu fraction was extracted with a solution of diethylene-triamine-penta-acetic acid (DTPA), CaCl_2_ 2H_2_O (0.01 M), and triethanolamine (0.1 M) at pH 7.3 with a 1:2 v/w soil:solution ratio, following the official standard methods of soil analysis (Italian Official Gazette n. 248, 1999). Total earthworms’ Cu concentration was measured after leaving the earthworms overnight to purge and euthanising them with 70% ethanol. Euthanised earthworms were oven-dried and ground as described by Wang et al. (2018), and each earthworm was then acid digested according to Tang et al. (2019).

All digested extracts (both from soil and earthworm) were analysed in triplicates using an inductively coupled plasma—optical emission spectrometer (Agilent 5800 ICP-OES, Agilent Technologies, Santa Clara, USA) according to EPA 6010D method (US EPA, 2014). For quality assurance and quality control purposes, blanks and laboratory control samples were employed at 5% rate.

For the SCGE test, three earthworms, one for each replicate, were collected at three incubation times, respectively at 2, 14 and 28 days. The SCGE, also known as comet assay, was performed on earthworms’ coelomocytes. The coelomocytes were collected, as described in Eyambe et al. (1991) with slight modifications, as previously described by De Bernardi et al. (2022a). Comet images were acquired in triplicate, and 300 comets per treatment were processed to calculate two major DNA damage index: tail length (TL) and tail intensity (TI) (Orlando et al. 2018; Tiano et al. 2005).

After coelomocites’ extrusion, the same earthworms from the initial (2 days) and final time (28 days) were sectioned, and their gut was used for the microbiome analysis. Each extruded earthworm was euthanised in 70% ethanol, and the midgut, spanning for 20 segments beyond the clitellum, was dissected using sterile equipment and stored at − 20 °C until molecular analyses.

Molecular analyses on the gut bacterial community were based on high throughput sequencing (HTS) of 16S rDNA amplicons. The total DNA collection was performed, as described by Swart et al (2020b) and Tang et al (2019). Genomic DNA was isolated using Soil DNA Isolation Kit (NORGEN Biotek, Canada) following the manufacturer’s protocol, and V3–V4 region of 16S ribosomal RNA (rRNA) gene was amplified using the universal primers 343F (5′-TACGGRAGGCAGCAG-3′) and 802R (5′-TACNVGGGTWTCTAATCC-3′), as previously described in detail (Bandini et al. 2021; Vasileiadis et al. 2015, 2012). The sequencing process was performed by Novogene UK (Cambridge, UK), using the TruSeq DNA sample preparation kit for amplicon preparation (REF 15026486, Illumina Inc., San Diego, CA). The Novaseq 6000 Illumina instrument (Illumina Inc, San Diego, CA) was used to obtain 250-bp paired-end reads. Illumina barcode demultiplexing and base calling were performed with the MiSeq Control Software version 2.3.0.3, RTA v1.18.42.0 and CASAVA v1.8.2 (Bortolini et al. 2016). Raw sequences were aligned with the ‘pandaseq’ script (Bartram et al. 2011) with a minimum overlap of 30 bp between read pairs and a maximum of two mismatches allowed. After the filtration, trim and denoising of the demultiplexed sequences of each sample, the chimeric sequences were recognised and removed using the QIIME™ 2 vsearch plugin to acquire the feature table of amplicon sequence variants (ASV). The QIIME™ 2 feature-classifier plugin was then applied to align the ASV sequences with a pre-trained Silva (trimmed to the V3–V4 region bound by the 338F/806R primer pair) to produce the taxonomy table. Thermal cycling conditions, primer concentrations and volumes are provided in Supplementary Table S1.

### Statistical analyses

In the avoidance test, the Fisher exact test with *α* = 0.05 was used to assess if the mean number of individuals at the end of the test in the treated soils was significantly lower than the mean number of earthworms found in the control (FC) soil. As reported by other authors (Natal‐da‐Luz et al. 2008), this test was set with the alternative “one-tailed" version, while in the case of dual control test, the “two-tailed” version was performed.

In the reproductive toxicity tests, due to non-normal data distribution, the significant differences between treatments were assessed using the Kruskal–Wallis test and Dunn’s post hoc test; the tests were performed using ‘dunn.test’ package version 1.3.5. The significant differences (*α* = 0.05) among Cu concentrations in both earthworms and soils were estimated using the Mann–Whitney-Wilcoxon non-parametric test.

Statistical analysis of avoidance and reproduction test was carried out with R software (R Core Team 2022).

For the genotoxicity test, the analysis of tail intensity and tail length was performed using the GraphPad Prism version 5 software. The Shapiro–Wilk test was applied to check data distribution that resulted not normal. Significant differences between the untreated vineyard soil (FC), and the other treatments were verified using the ANOVA One-Way test and Dunnet’s post hoc test.

Statistical analyses on sequencing data were performed with R (http://www.R-project.org/) supplemented with Vegan package (Dixon 2003) and MicrobiomeAnalyst (Chong et al. 2020).

## Results and discussions

### Avoidance and reproductive outputs

Validity criteria were fulfilled for the avoidance tests since no earthworms died, and their distribution between the two sections was approximately the same in the dual-control tests carried out with the ART soil (47:53%) and FC soil (52:48%) (data not shown). There was indeed no significant avoidance behaviour (Fisher test *p*-value > 0.05) when the same soil was placed on each side of the test vessel.

The effects of the three spiked Cu concentrations on the avoidance behaviour are reported in Table 1 as net response (*NR*) values; no earthworm escaped from soils or died during the exposure period in the avoidance test. Table 1 summarises the results of the reproductive activity in terms of cocoons and juveniles hatched as well. A slight mortality was measured only in the trial with 220 mg/kg.

**Table 1:** Observations on earthworms’ avoidance and reproduction outputs in vineyard soil spiked with different Cu sub-lethal concentrations (*mean* ± *SD*)

Parameters	Treatments				
	ART	FC	110	165	220
Net response (%)	-	-	52.0 ± 9.8*	76.0 ± 14.9***	84 ± 14.8***
Cocoons, number	52.0 ± 7.5^b^	51.3 ± 2.5^b^	42.3 ± 7.6^ab^	35.3 ± 7.6^ab^	21.3 ± 4.6^a^
Juveniles, number	115.0 ± 9.8^b^	109.6 ± 9.5^b^	61.3 ± 8.9^ab^	64.0 ± 9.0^ab^	34.3 ± 10.1^a^
Growth rate (%)	13.2 ± 4.1^b^	11.5 ± 4.9^ab^	7.5 ± 1.5^ab^	10.3 ± 3.2^ab^	2.5 ± 1.2^a^
Mortality (%)	-	-	-	-	13.3 ± 9.4

According to Dunn’s Kruskal–Wallis multiple comparisons, treatments with different lowercase letters were significantly different (*α-level* = 0.05). Asterisks on *NR* values refer to statistical differences by Fisher exact test between the number of earthworms in the two sections of the avoidance test chamber (* *p* < 0.05; ** *p* < 0.01; *** *p* < 0.001; **** *p* < 0.0001).

A positive *NR* indicated an avoidance of the contaminated substrate, whereas a negative value indicated an attraction to the tested contaminant (Gainer et al. 2022; Xu et al. 2020). According to the standard protocol (ISO-17512–1, 2008), the soil has limited habitat function if the positive *NR* is higher than 70%; this occurs when at least 80% of earthworms avoid the treated substrate.

A positive net response (*NR*) value was measured in all trials. A dose–response behaviour was detected in the earthworms’ avoidance; as contamination increased, a significantly higher number of earthworms preferred less contaminated soil (FC). At the two highest Cu concentrations tested (165 and 220 mg/kg), more than 80% of earthworms (respectively 88 and 90%, data not shown) preferred the unspiked soil, indicating that soils with these two Cu doses represent a limiting habitat. In Xing et al. (2017), the habitat limit was exceeded by 128 mg/kg of copper in the soil, and a 100% *NR* was measured at the 160 mg/kg dose (in our case the *NR* values are 76 and 84% for 165 and 220 mg/kg, respectively).

Similar results were also obtained with other earthworm species. Lukkari et al. (2005), found that *Aporrectodea tuberculata* begins to avoid Cu-contaminated soil at around 50 mg/kg, and from 79 mg/kg onwards, the habitat limit threshold was exceeded. In a recent work by Renaud et al. (2022), avoidance test results were expressed as contaminant concentrations (AC) that could limit 50 and 80% of the individuals, namely AC50 and AC80; in that work, it was found that the Cu AC50 and AC80 for *Eisenia andrei* in the tested soil were 49.5 mg/kg and 112 mg/kg, respectively. In the present study, already at 110 mg/kg of Cu contamination, 76% of earthworms avoided the soil (*NR* of 52%), while at 165 mg/kg, the avoidance reached 88% (*NR* of 76%).

The analyses of the growth rate (*GR*) during the reproduction test highlighted significant differences from the ART soil with very low presence of copper were noted only at the end of 28 days for earthworms subjected to the highest Cu concentration, but this trend could be explained by the different substrate. As it can be observed from the data summarised in Table 1, the dose-dependent trend is evident for all the reproduction outputs, although the highest dose caused substantial alterations in earthworms’ reproductive outcomes compared to the unspiked soil (FC).

Reproduction assays are considered very sensitive as compared to other indicators (such as mortality or growth) when studying the effects of xenobiotics at sub-lethal doses (Ge et al. 2018; Hund-Rinke et al. 2003; Van Gestel 1992; Žaltauskaitė and Sodienė, 2010). More in detail, a study conducted by Žaltauskaitė and Sodienė (2010) revealed that reproduction tests are more suitable in studies with metals because the number of coccons is more sensitive than other endpoints with this type of toxicants.

In the present work, a cocoon production inversely proportional to the Cu soil concentration was observed, in agreement with other literature evidence (Clasen et al. 2021; Duan et al. 2016; Tatsi et al. 2018). For example, Owojori et al. (2010) found that a soil Cu concentration above 80 mg/kg resulted in a significant decrease of cocoon production as compared to the control. Scott-Fordsmand et al. (2000) found for a natural soil a Cu EC_50_ 210 mg/kg for cocoon production of *E. fetida*, while in the experiment conducted by Zhou et al. (2013), it was observed a dose-dependent trend in copper toxicity in the reproduction outputs and a halving in the number of cocoons at the highest dose (200 mg/kg) as compared to the control. Accordingly, in the present study, at 220 mg/kg Cu concentration, cocoon counts halved as compared to those measured in ART and FC.

The trend of the earthworm weights during the first 28 days of the reproductive experiment is reported in Figure S1. After the first week, an evident weight decrease in the 220 mg/kg treatment was observed, and although a partial recovery was measured in the following weeks, reaching weights similar to those measured in all the other theses (at 14 days of exposure), the weight gain at the end of the test was almost null. On the contrary, for the 165 mg/kg treatment, a positive trend of weight gain was evident (Figure S1).

It seems that up to Cu concentrations ranging from 110 to 165 mg/kg, earthworms should be able to adapt and increase their weight. The 220 mg/kg dose, despite the initial recovery, leads to a weight loss more evident as compared to the other theses, while the average earthworm weights in both the uncontaminated soil (ART), unspiked soil and 110 mg/kg treatment were not significantly different.

Earthworm trophic activity decreased following high Cu exposure, as reported by other authors (Tatsi et al. 2018). Similar biomass trends with a weight gain up to a critical Cu concentration followed by a significant weight loss at higher concentrations was also shown in other experiments (De Bernardi et al. 2022b; Clasen et al. 2021; Owojori et al. 2010); these occurrences are common in the presence of toxicants and are clearly associated to hormesis phenomena. In the earthworm reproduction test by Lukkari et al. (2005) conducted on a high organic content substrate (~ 7%), the biomass of *A. tuberculata* was increased at intermediate Cu doses ranging from 53 to 79 mg/kg, while it decreased at the highest tested concentrations ranging from 119 to 268 mg/kg. In other studies, soil Cu concentrations between 100 and 300 mg/kg caused decreases in earthworm biomass (Malecki et al. 1982; Svendsen and Weeks 1997).

Variability in the no-observed-adverse-effect level is undoubtedly linked to various factors such as substrate properties, earthworm species (Calisi et al. 2011; Duan et al. 2016; Karimi et al. 2021), earthworm development stage and Cu formulation administered (Gainer et al. 2022; Wang et al. 2018). In Helling et al. (2000), for example, adverse effects were already measured on the earthworms’ growth at 8.92 mg/kg soil Cu, but it should be noted that freshly hatched earthworms were used instead of clitellate earthworms, as in the present study. In fact, it is known that young earthworms are more sensitive to xenobiotics than adult ones (Spurgeon and Hopkin 1996).

Overall, our results relative to reproductive indexes are in agreement with previous observations reported in the literature (Gao et al. 2016), highlighting a correlation between the impairment of earthworms’ reproductive activity and soil Cu concentration at values higher than 120 mg/kg, which in the present work were between 165 and 220 mg/kg.

### Genotoxicity and gut microbiome results

#### Copper measurements

**Fig. 1:**
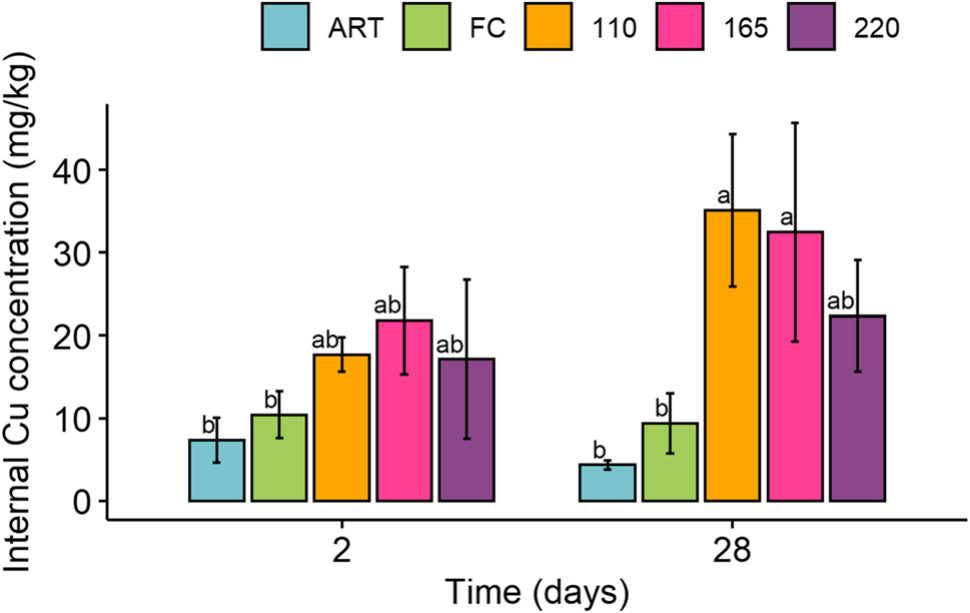
Copper concentrations in earthworms (mg Cu on kg earthworms dry weigth) exposed for 28 days to vineyard soil spiked with different Cu concentrations. According to Dunn’s Kruskal–Wallis multiple comparisons, treatments with different lowercase letters were significantly different (*α-level* = 0.05). Treatment codes’ legend: FC, unspiked soil with 55 mg/kg Cu; 110, soil Cu concentration of 110 mg/kg; 165, soil Cu concentration of 165 mg/kg; 220, soil Cu concentration of 220 mg/kg; ART, artificial uncontaminated soil

Following 28 days of exposure, Cu concentrations in earthworms exposed to soil with 110 and 165 mg/kg Cu were significantly higher than in individuals grown both on uncontaminated artificial soil (ART) and unspiked control soil (Fig. 1).

On the contrary, at the highest concentration tested (220 mg/kg), Cu concentration in earthworm tissues decreased. This non-linear dose–response may result from a homoeostatic reaction triggered by earthworms above a particular Cu soil threshold to counteract excessive tissue accumulation. Similar behaviours were reported by other authors (Gao et al. 2016; Nahmani et al. 2007; Natal-da-Luz et al. 2011; Richardson et al. 2020; Spurgeon and Hopkin 1999). In fact, it is not surprising that earthworms have developed systems to modulate Cu absorption and excretion in order to regulate their tissue content with low bioaccumulation levels, being Cu an important trace element (Ruiz et al. 2009). On the contrary, not essential elements such as Pb, Cd and As do not seem to be regulated (Garau et al. 2022; Porfido et al. 2022).

Taking into account that at 28 days the earthworms exposed to the highest concentration of Cu had a significantly lower average weight compared to the other treatments (Figure S1), this may be proposed as an index of compromised metabolism and lower trophic activity, which also contribute to the reduced intake and therefore accumulation of the element.

Total and the potentially bioavailable Cu concentrations in soils were also analysed in order to better understand and contextualise the potential bioaccumulation in earthworms during the tests. The measurements were performed both in the presence and absence of earthworms to isolate also potential contributions of the earthworms to changes in bioavailable Cu (Table 2). According to the Mann–Whitney-Wilcoxon test, no significant differences in terms of potentially bioavailable (DTPA-extractable) Cu were measured within the same treatment between the initial (2 days) and final (28 days) exposure times; similarly, within the same exposure time, no significant differences were recorded between the treatments in earthworms’ presence or absence.

**Table 2:** Total and potentially bio-available Cu concentrations (mg/kg) in vineyard soils incubated for 28 days in both earthworms’ presence and absence

Treatments	Nominal soil Cu (mg/kg)	Earthworms’ presence	Total soil Cu (mg/kg)		DTPA-extractable Cu (mg/kg)	
			2 days	28 days	2 days	28 days
ART	0	−	3.09 ± 0.37	1.27 ± 0.54	0.14 ± 0.09	0.10 ± 0.03
		+	3.46 ± 0.40	3.99 ± 0.57	0.11 ± 0.01	0.11 ± 0.03
FC	55	−	53.12 ± 3.03	48.40 ± 3.52	3.82 ± 0.23	4.56 ± 0.42
		+	61.51 ± 0.68	57.02 ± 0.97	4.46 ± 0.29	3.79 ± 0.30
110	110	−	104.13 ± 8.44	95.96 ± 4.93	24.4 ± 0.75	20 ± 1.73
		+	101.05 ± 6.46	90.71 ± 4.61	23.1 ± 1.25	15.9 ± 1.49
165	165	−	143.48 ± 3.53	129.09 ± 5.50	50.4 ± 7.15	34.9 ± 2.15
		+	149.66 ± 10.00	132.91 ± 11.00	45.8 ± 2.11	25.2 ± 2.85
220	220	−	191.35 ± 4.85	180.82 ± 13.9	53.0 ± 9.97	52.5 ± 4.52
		+	178.68 ± 11.0	186.04 ± 11.50	66.5 ± 2.70	38.6 ± 8.72

Likewise, in Fujii and Kaneko (2009), the earthworm’s activities did not significantly influence the values of potentially bioavailable Cu (DTPA extractable) in a 28 day-test conducted with both freshly-spiked Cu and aged-contaminated soils. In fact, according to literature, 28 days are generally required in earthworms to reach copper homeostasis at the organism’s level (Kennette et al. 2002; Spurgeon and Hopkin 1999). In contrast, according to Dandan et al. (2007) for earthworm species different from *E. fetida*, such as *Metaphire guillelmi*, an increase of the Cu DTPA-extractable fraction was observed.

Furthermore, the bioaccumulation factor (BAF) was also measured to estimate the potential occurrence of bioaccumulation. The bioaccumulation factor (BAF) is defined as the ratio between the earthworm’s Cu concentration to the total Cu in the soil (Richardson et al. 2020).

In the present work, the BAFs had values always below the unit, namely 0.2, 0.4, 0.2 and 0.1, for the FC, 110, 165 and 220 treatments, respectively, indicating that Cu is not bioaccumulated in earthworms; similar conclusions were drawn in a study comparing different forest soil and earthworm species conducted by Richardson et al. (2015).

### Genotoxic effects on *E. fetida*

**Fig. 2:**
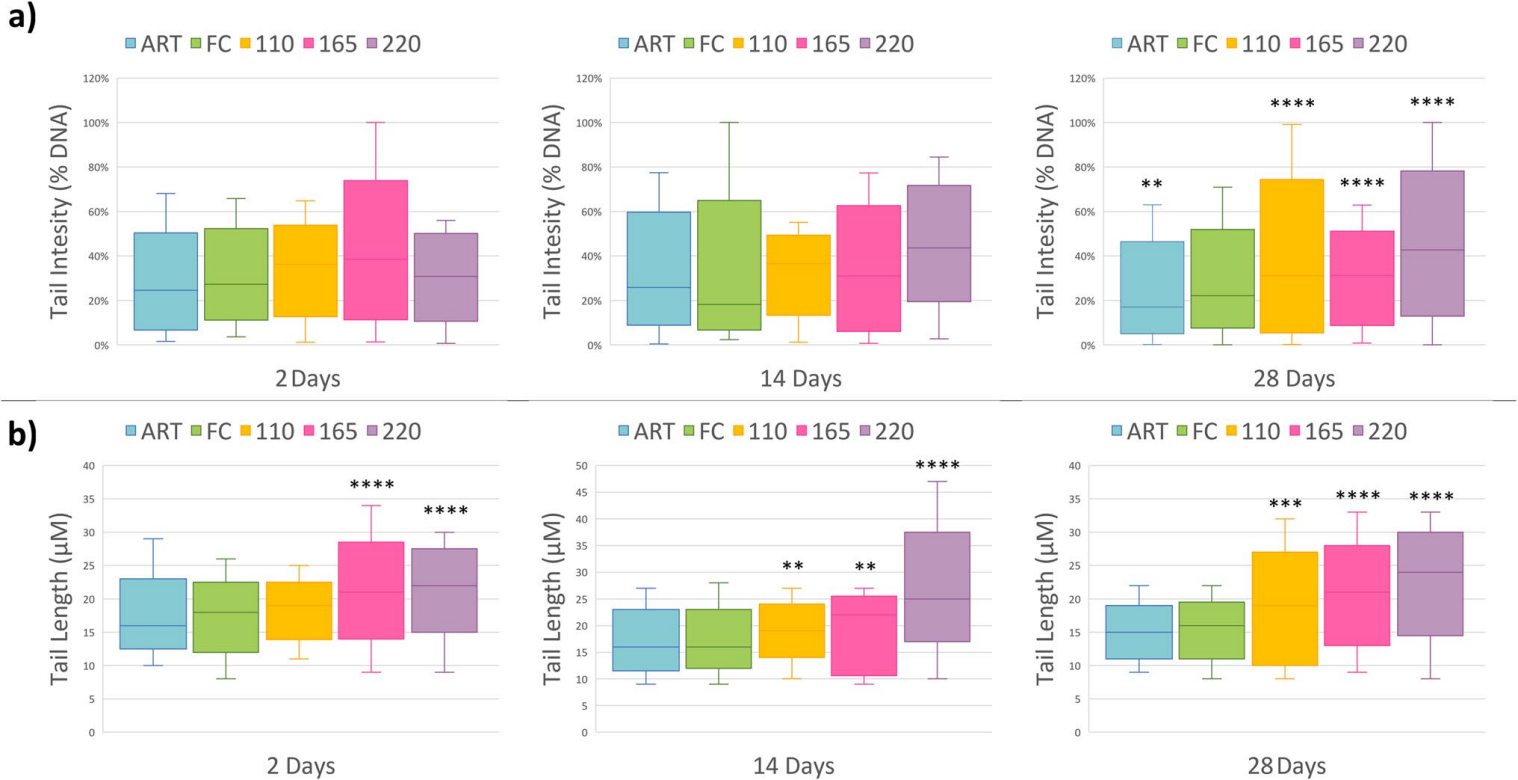
Tail intensity **a** and tail length **b** in coelomocytes from earthworms exposed for three different times to vineyard soils contaminated with Cu to different degrees. Asterisks refer to differences between each treatment and FC soil calculated by one-way ANOVA using multicomparison Dunnet’s test (* *p* < 0.05; ** *p* < 0.01; *** *p* < 0.001; **** *p* < 0.0001). Codes’ legend: FC, unspiked soil with 55 mg/kg Cu; 110, soil Cu concentration of 110 mg/kg; 165, soil Cu concentration of 165 mg/kg; 220, soil Cu concentration of 220 mg/kg; ART, artificial uncontaminated soil

DNA damage in earthworms’ coelomocytes was summarised in Fig. 2. Figure 2 a reported the tail intensity that indicates the estimated percentage of damaged DNA, while Fig. 2 b reported the tail length that outlines the extent of the DNA damages measured as µM of degraded DNA migrated.

After 2 days of exposure, no significant damages in terms of TI were detected in any treatment compared to FC, even if the median value of the ART soil (25%) was lower than all other treatments. At the same exposure time, the TL, which is a more sensitive parameter, particularly at low DNA degradation, showed significant damages (*p* < 0.0001) at the two higher Cu doses (165 and 220 mg/kg) compared to FC. At the intermediate (14 days) exposure time, there were still no significant differences in terms of TI, even if the median values of the spiked treatments remained higher than both the ART and FC. On the contrary, TL index showed a significant dose-dependent damage trend starting from the 110 mg/kg Cu concentration..

Likewise, in the study by Zheng et al. (2013), copper-induced DNA damages were visible already after 2 days and remained significant until the end of the test (28 days). Despite this, the authors highlighted the triggering of a compensatory mechanism that led to a DNA-damage downward trend from 14 days onwards and which was possibly associated with the activation of antioxidant defences against reactive oxygen species by means of metal compartmentalization processes. The different results as compared to ours observed could be because Zheng et al. (2013) worked with soils contaminated by multiple metals at higher doses, whereas in the present study the only studied contaminant was Cu.

Significant *TI* damage was measured at prolonged exposure (28 days) that rose with the Cu doses administered, except for ART, where the *TI* values were significantly lower than FC. The TL damages at 28 days were also confirmed for all the spiked soils. A positive correlation between the increase of coelomocytes’ DNA damage and exposure time was also found by Yan et al. (2021), where earthworms were exposed to insecticides and metals combined pollution.

The genotoxicity test with the comet assay allowed the detection of early damage (in terms of TL) already at 2 days of exposure, which would not be possible with other tests. This method, developed in the 90 s for biomonitoring in the medical field, has been implemented for a long time and is used for its versatility with eukaryotic and prokaryotic organisms and different types of cells and matrices. The test provides robust, reliable sensitive and quantitative results compared to classic genotoxic tests such as alkaline elution, sister chromatid exchanges, micronucleus assays and chromosomal aberration assay (Collins et al. 2023; de Lapuente et al. 2015; Jiang et al. 2023; Vischetti et al. 2020).

### Effects on gut bacterial community

**Fig. 3:**
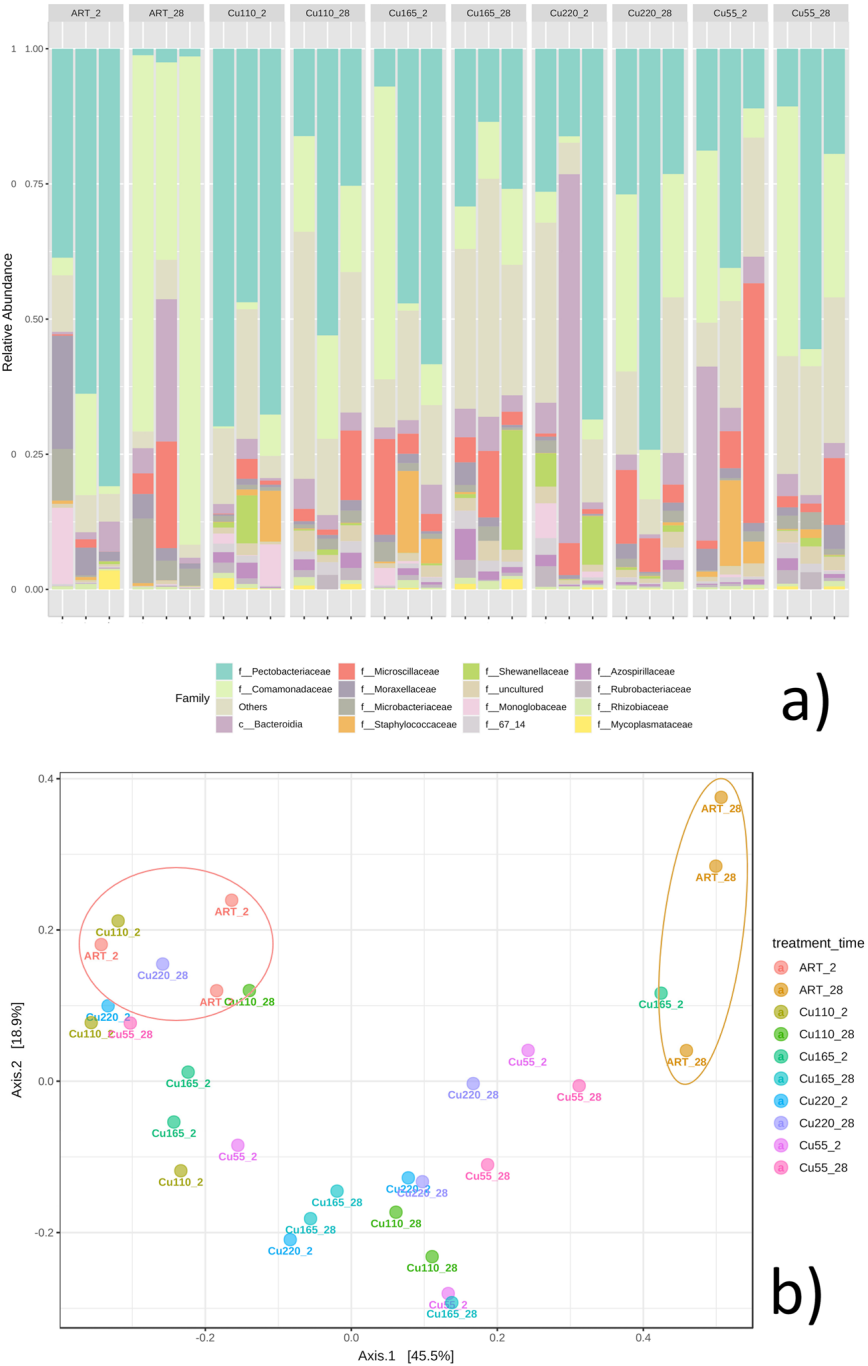
Gut bacterial community structure of earthworms exposed to Cu-contaminated vineyard soils at different concentrations. **a** Relative abundance of the top 15 bacterial families hosted in *E. fetida* gut exposed to different Cu contamination. Less abundant taxa were included in “Others” group; **b** principal component analysis (PCA) performed on the total bacterial ASVs’ relative abundance. ART sample clusters are highlighted with circles in Fig. 3 b. Treatments codes’ legend: Cu55, unspiked soil with 55 mg/kg Cu; Cu110, soil Cu concentration of 110 mg/kg; Cu165, soil Cu concentration of 165 mg/kg; Cu220, soil Cu concentration of 220 mg/kg; ART, artificial uncontaminated soil

The bacterial community was carefully analysed in terms of diversity and abundance both at the beginning and the end of the test. After quality control, a total of 500,000 high-quality 16S rRNA sequences were obtained from all samples, with each sample ranging from 15,000 to 20,000 sequences. The results are outlined both in Fig. 3, and in supplementary figures S2, S3 and S4. The 16S amplicon sequencing analyses showed distinct profiles in the gut microbiome of *E. fetida* exposed to soil with different degrees of Cu contamination (Fig. 3).

**Fig. 4:**
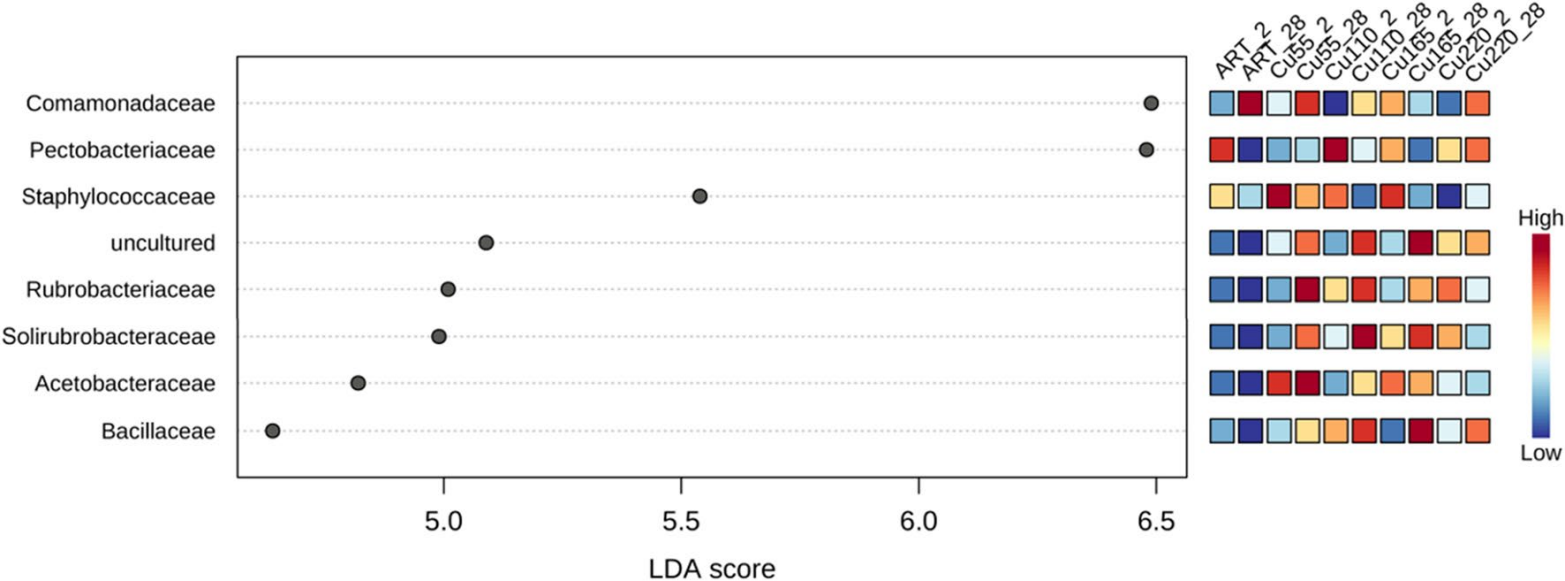
The effect size analysis of the treatment groups was conducted using linear discriminant analysis (LDA) (*p*-value < 0.05). An LDA score > 4 indicates statistically significant differences of the taxa between groups. The abundance of bacterial families has been normalised for the total abundance of samples, and the most significant features have been presented

We observed intricate dynamics in the microbial communities of the guts of earthworms (*E. fetida*) in response to varying concentrations of copper (Cu) and over different sampling periods. Notably, families such as *Rubrobacteriaceae*, *Solirubrobacteraceae*, *Acetobacteriaceae*, *Bacillaceae* and *Staphylococcaceae* exhibited distinct abundance patterns, which are indicative of the complex interplay between environmental Cu levels and microbial ecology. The LefSe analysis reported in Fig. 4 revealed a less pronounced abundance of certain families like *Rubrobacteriaceae* and *Solirubrobacteraceae* in ART (artificial soil) compared to Cu-spiked soil, suggesting a potential facilitative role of Cu in promoting their growth or survival.

However, given the different characteristics of artificial and environmental soil samples, such conclusions cannot be confidently drawn, and further research is needed. This observation aligns with the distinct separation of microbial compositions between ART and Cu soils at the 28-day mark, as revealed in the PCA analysis (Fig. 3). The higher abundances of these families’ in the gut microbiome of *E. fetida* at 28 days further underscore the impact of prolonged Cu exposure on microbial dynamics. In particular, the *Bacillaceae* family, known for its role in organic matter decomposition and soil health (Mandic-Mulec et al. 2016), demonstrated a time-dependent increase with higher abundances at 28 days in gut samples spiked with Cu, with the highest levels observed in the Cu_165_28 sample. This trend highlights the adaptability of this family to Cu exposure and its potential role in maintaining gut functionality and soil processing efficiency. Such an adaptation is crucial for the ecological role of *E. fetida* in soil nutrient cycling and overall soil health. Conversely, the fluctuation in abundance of *Acetobacteriaceae*, with a higher presence in lower Cu concentrations and a decrease at higher levels, points to a sensitivity to Cu that could impact their ecological function in the gut ecosystem. The effect of environmental chemical exposure to Cu nanoparticles was previously linked to a reduction in *Acetobacter* in the gut environment of *Drosophila melanogaster* (Rosenfeld 2017). *Acetobacteriaceae* are indeed involved in nutrient cycling and include a nitrogen fixing microrganism (Reis and Teixeira 2015), thus emphasizing the relationship between metal exposure and microbial community structure. Furthermore, our findings on the presence of *Pectobacteriaceae*, a family that includes phytopathogenic bacteria (Van Gijsegem et al. 2021), and *Comamonadaceae*, producers of essential enzymes for vermicomposting (Budroni et al. 2020), highlight the broader ecological consequences of these microbial shifts. The three most abundant families found were *Pectobacteriaceae*, *Comamonadaceae* and *Microscillaceae*. The latter family is a producer of chitinase, cellulases and hemicellulases, essential enzymes during the vermicomposting process and for gut homeostasis. A reduction in *Comamonadaceae* caused by the treatment can indicate impaired gut functionality in *E. fetida*. Jiang et al. (2021) also observed a similar effect in the presence of treatment with metal oxides, among which copper oxide (CuO) was present, for *Comamonadaceae*. However, the CuO presence was not detrimental for the *Microscillaceae* family, which was confirmed by our results too. Furthermore, *Comamonadaceae* are also thought to be important plant associates in the control of phytopathogens because of their enzymatic activities. Changes in the abundances of some bacterial groups in our study can also be considered as potential indicators of the ability of earthworms to alter the structure of the soil bacterial community as the abundance of *Proteobacteria* and *Bacteroidota* (of which most abundant families found in the gut microbiomes in our study also belong) were closely related to earthworm activities in the soil in a study conducted by Sofo et al. (2023). These observed trends in microbial abundance, influenced by Cu exposure, reflect not only the direct impacts on specific bacterial families but also broader ecological implications. These include alterations in the soil bacterial community structure, potential effects on plant health and the sustainability of agricultural practices. Understanding these microbial dynamics is vital for assessing the environmental impact of heavy metal contamination and developing strategies for soil and ecosystem management. Further research is needed to confidently discern the facilitative role of Cu in promoting the growth or survival of certain microbial families, given the different characteristics of artificial and environmental soil samples.

## Conclusions

The current research contributes to the understanding of Cu ecotoxicity in agricultural soil ecosystems. The results here gathered hold particular relevance for organic viticulture, where Cu pesticides are among the few ones allowed for plant defence; these results can thus provide new and relevant information that could stimulate and produce advancements at the regulatory level.

In this work, a new array of complementary ecotoxicological tests together with the classical ones were used with the aim of obtaining a more accurate assessment of the potential impacts of contaminants such as Cu on agricultural ecosystems. Notably, analysis of the avoidance behaviour showed the highest sensitivity of earthworms to copper concentrations in soil; above 110 mg/kg, the soil habitat limit was exceeded, over these values; there were significant responses in terms of earthworms’ biomass alterations as well. Specifically, earthworms were stressed but could regain their weight as long as the 165 mg/kg Cu dose was not exceeded, while already at 220 mg/kg, the weight loss and the impaired reproductive activity were irreversible.

The SCGE genotoxicity tests allowed to identify and to measure relevant DNA damages as early as after 2 days of exposure at 165 mg/kg; dose and time-dependent increasing trends were measurable for all the spiked treatments. Damages, although of minor entity, were already identifiable in the unspiked soil characterised by 55 mg/kg Cu concentration. The results of *E. fetida* gut 16S amplicon sequencing showed distinct patterns of abundance in families such as *Rubrobacteriaceae*, *Solirubrobacteraceae*, *Acetobacteriaceae*, *Bacillaceae* and *Staphylococcaceae*, highlighting the complex interplay between environmental Cu levels and microbial ecology. Notably, the time-dependent increase in *Bacillaceae* abundance suggests its adaptability to Cu exposure, essential for maintaining gut functionality and soil processing efficiency. Conversely, the sensitivity of *Acetobacteriaceae* to higher Cu levels underscores the nuanced impact of metal exposure on microbial community structure and ecological function.

The results indicate how a holistic approach that considered innovative tools beside classic ecotoxicological endpoints can provide more detailed and sensitive information on the effects of Cu pollution on non-target soil organisms such as the earthworm *E. fetida*.

### Supplementary information

Below is the link to the electronic supplementary material.

Supplementary file1 (DOCX 942 kb)

## Data Availability

Data supporting the findings of this study are available upon request from the corresponding author.
