# Supplementary material for: Copper toxicity on Eisenia fetida in a vineyard soil: a combined study with standard tests, genotoxicity assessment and gut metagenomic analysis
Source: Environ Sci Pollut Res Int. 2024 Jan 19;31(9):13141–54. doi: 10.1007/s11356-024-31946-6 (PMC10881645; doi:10.1007/s11356-024-31946-6)
Supplement: Supplementary file 1 — Supplementary file1 (DOCX 942 kb) [file 11356_2024_31946_MOESM1_ESM.docx]

**Supporting Information for:**

**Copper toxicity on *Eisenia fetida* in a vineyard soil: a combined study with standard tests, genotoxicity assessment, and gut metagenomic analysis**

Enrica Marini^a^, Arianna De Bernardi^a*^, Francesca Tagliabue^a^, Cristiano Casucci^a^, Luca Tiano^b^, Fabio Marcheggiani^b^, Filippo Vaccari^c^, Eren Taskin^c^, Edoardo Puglisi^c^, Gianluca Brunetti^a,d^ and Costantino Vischetti^a^

^a^ Department of Agricultural, Food and Environmental Sciences, Polytechnic University of Marche, Via Brecce Bianche, 60131, Ancona, Italy;

^b^ Department of Life and Environmental Sciences, Polytechnic University of Marche, Via Brecce Bianche, 60131, Ancona, Italy;

^c^ Department for Sustainable Food Process, Faculty of Agriculture, Food and Environmental Sciences, Catholic University of Sacred Heart, Via Emilia Parmense 84, 29122, Piacenza, Italy;

^d^ Future Industries Institute, University of South Australia, Mawson Lakes Boulevard, South Australia, SA5095, Australia;

**Supplementary Table 1 (S1).** PCR reaction mixtures and thermal profiles for different target genes used.

| Target Gene | Reaction Mix | Volume (µL) | Step 1 |
| --- | --- | --- | --- |
| 16s rRNA  1^st^ step | Phusion Flash High-Fidelity Master Mix | 12.5 | 95 °C - 5 min  22x $\left\{ \begin{aligned} 95^{\circ}C 30s \\ 50^{\circ}C 30s \\ 72^{\circ}C 30s \end{aligned} \right.$  72 °C 10 min. |
|  | Nuclease free water | 8 |  |
|  | DNA template (1ng/μL) | 2 |  |
|  | Primer 343F (10µM)  (5′-TACGGRAGGCAGCAG-3′) | 1.25 |  |
|  | Primer 802R (10µM)  (5′-TACNVGGGTWTCTAATCC-3′) | 1.25 |  |
| 16s rRNA  2^nd^ step | Phusion Flash High-Fidelity Master Mix | 12.5 | 95 °C - 5min  10x $\left\{ \begin{aligned} 94^{\circ}C 30s \\ 50^{\circ}C 30s \\ 72^{\circ}C 30s \end{aligned} \right.$  72 °C 10min. |
|  | Nuclease free water | 8 |  |
|  | 1st Step Amplicons | 1.25 |  |
|  | Tagged Primer 343F (10µM)  (5′-TACGGRAGGCAGCAG-3′) | 1.25 |  |
|  | Primer 802R (10µM)  (5′-TACNVGGGTWTCTAATCC-3′) | 1.25 |  |


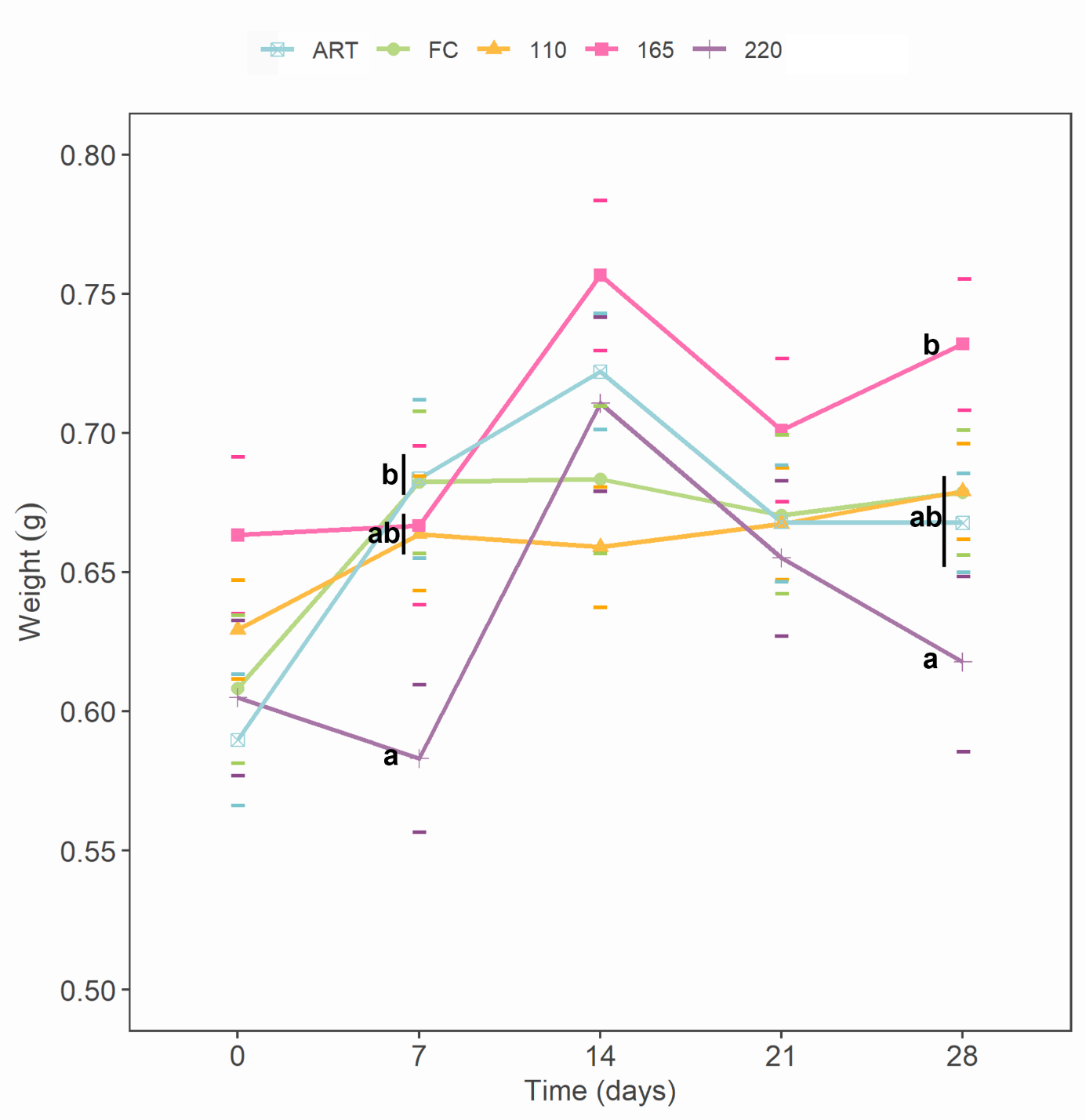


**Figure S1. *E. fetida*** **weights trends during the reproduction experiment in vineyard soil spiked with different Cu concentrations.** According to Dunn's Kruskal–Wallis multiple comparisons, treatments at the same time with different lowercase letters were significantly different (*α-level*=0.05). The horizontal bars represented the standard error's upper and lower limit values. Treatments codes legend: **FC**, unspiked soil with 55 mg/kg Cu; **110**, soil Cu concentration of 110 mg/kg; **165**, soil Cu concentration of 165 mg/kg; **220**, soil Cu concentration of 220 mg/kg; **ART**, artificial uncontaminated soil.


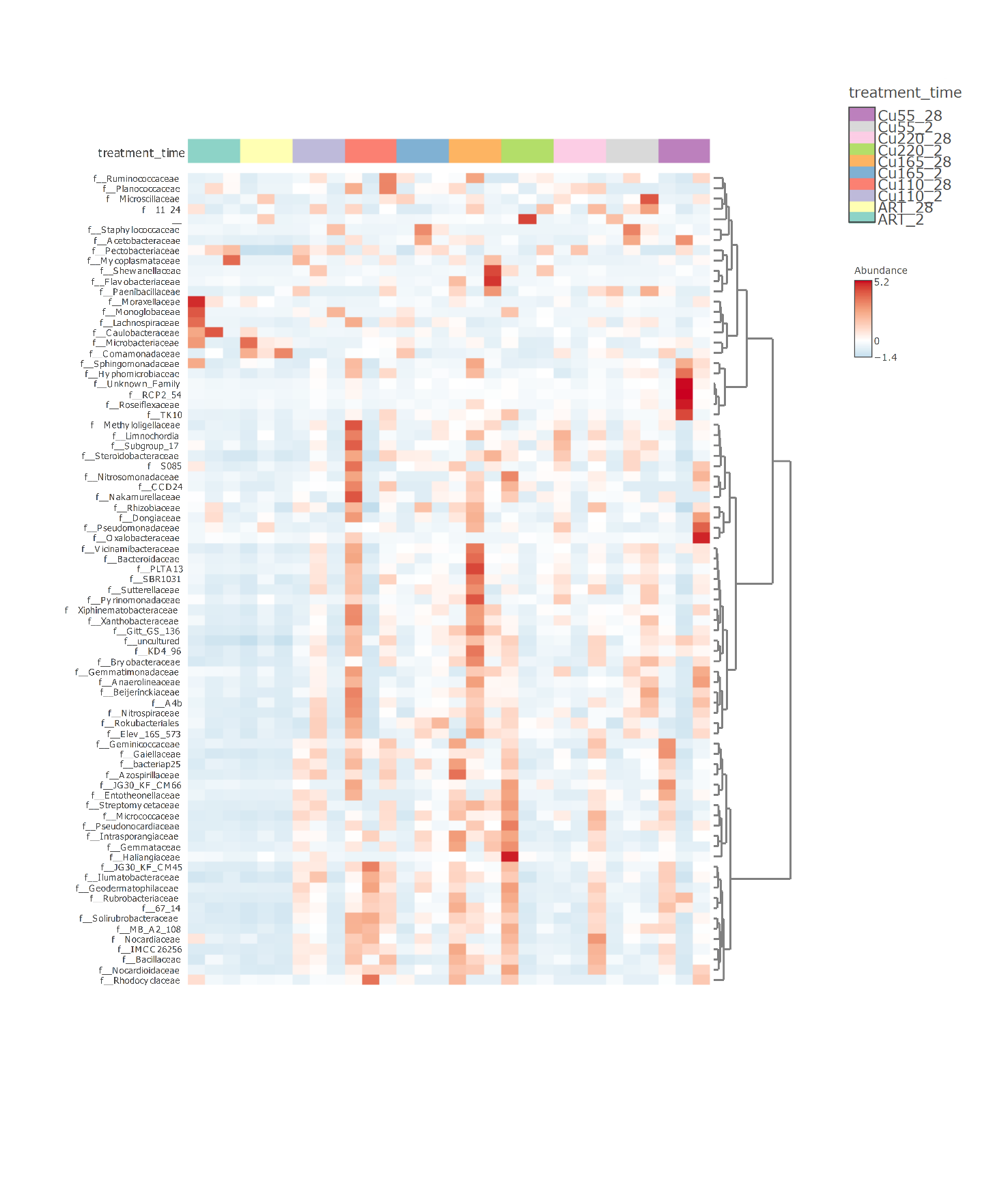
**Figure S2.** **Modulated families Clustering heatmap.** A heatmap showing the different bacterial families in *E.fetida* gut following different treatments and sampling times. Higher abundance of specific families is indicated with the colour red. Similarity in behaviour of the different families in terms of abundance was measured with Euclidean distance method and clustered with average linkage method.
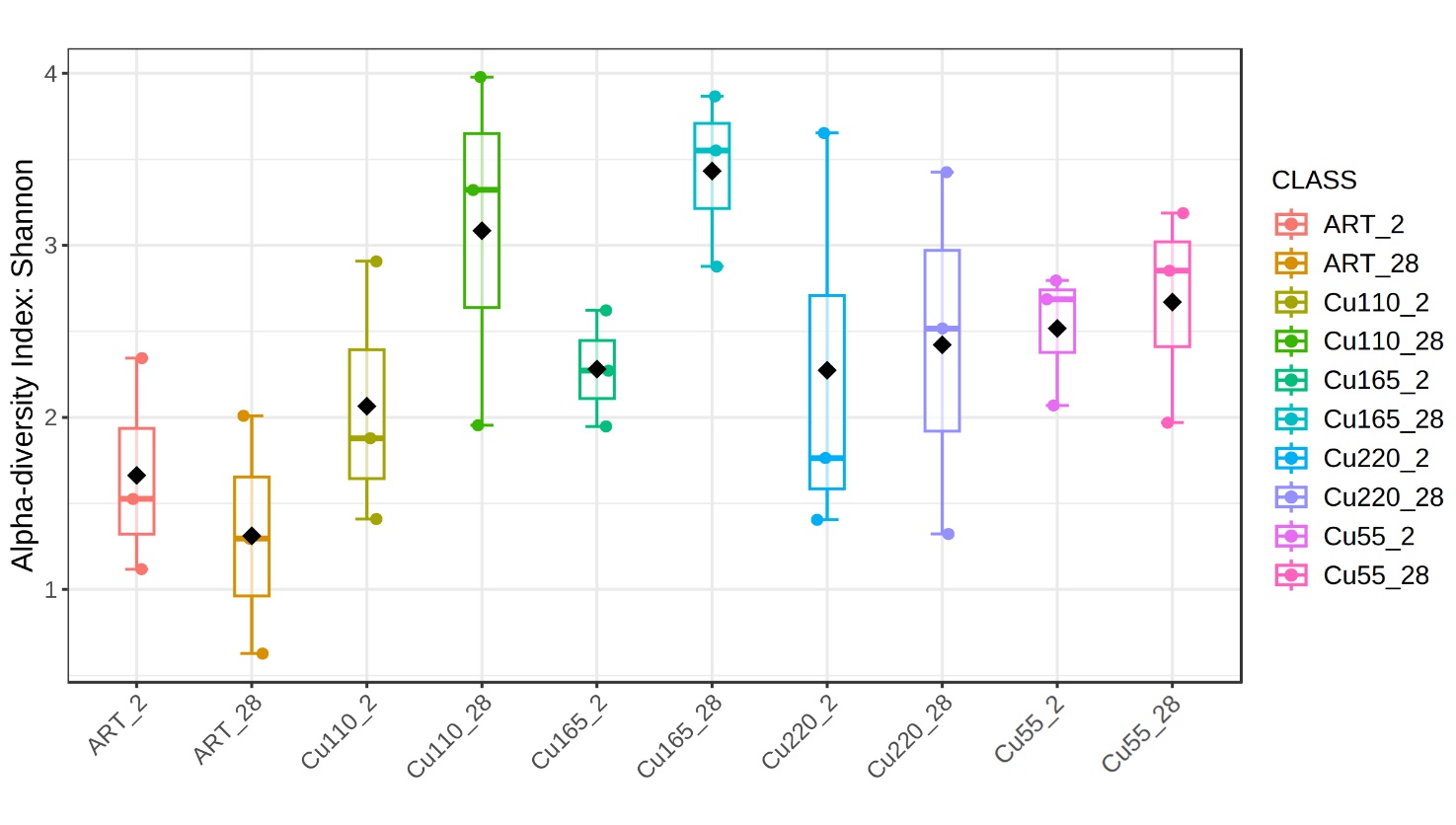
 **Figure S3.** **Alpha diversity plot.** Biodiversity of bacterial communities, as affected by the different Cu treatments and times, according to Shannon diversity index.


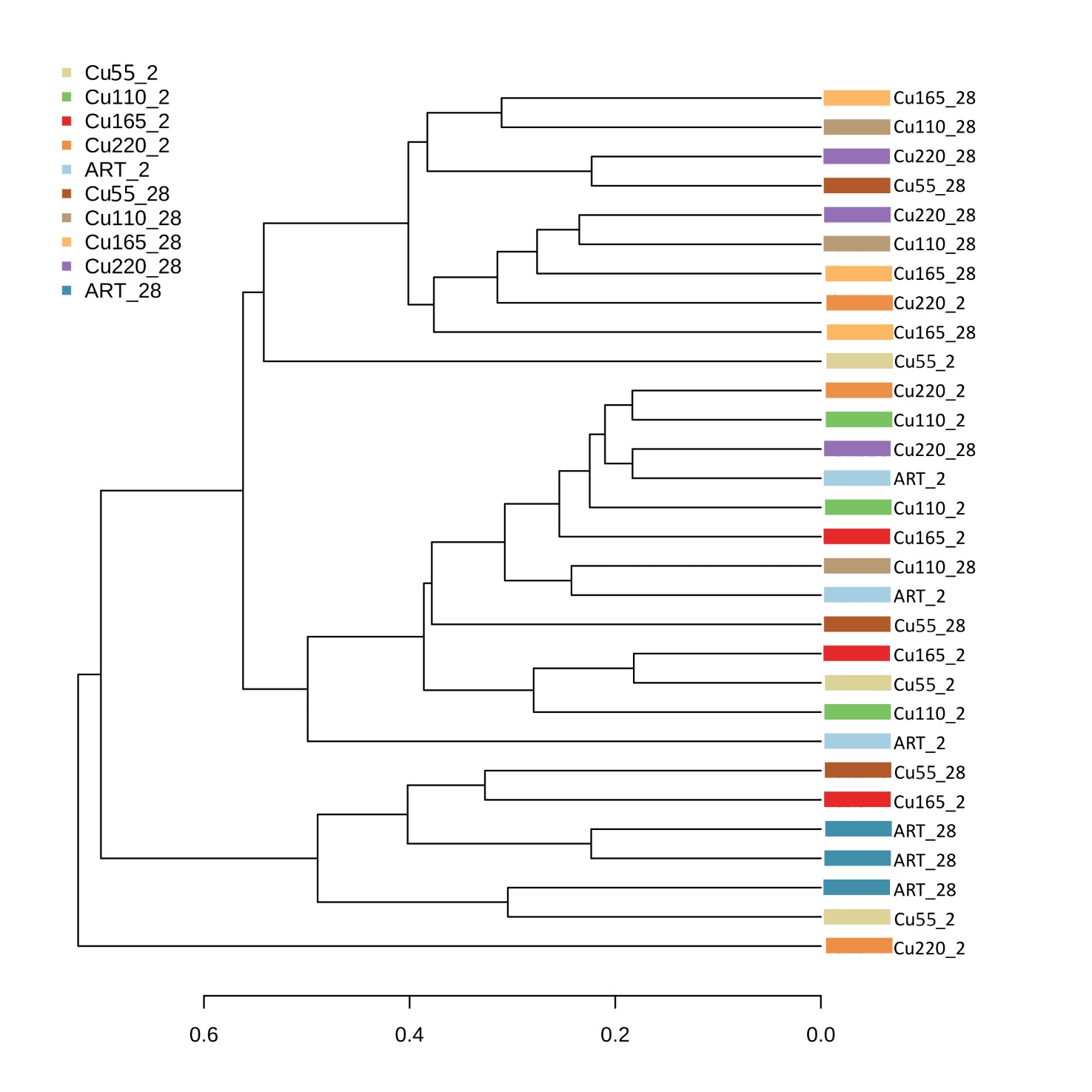


**Figure S4.** **Hierarchical cluster of the bacterial communities.** Feature-level dendrogram analyses showing the similarity between different samples. Distance measure was based on Bray-Curtis Index and clustering method was based on Average Linkage.
